# Rare missense variants in *NECTIN1* alter local protein structure and may contribute to non-syndromic cleft lip with or without palate

**DOI:** 10.3389/fgene.2026.1894075

**Published:** 2026-08-13

**Authors:** Ana Luiza Meneguci Moreira Franco, Deborah Antunes, Ana Carolina Proença da Fonseca, Ana Clara Rodrigues Moreira Gomes, Ana Carolina Ramos Guimarães, Ieda Maria Orioli, Flavia Martinez de Carvalho

**Affiliations:** 1 Laboratório de Epidemiologia de Malformações Congênitas (LEMC), Instituto Oswaldo Cruz, FIOCRUZ, Rio de Janeiro, Brazil; 2 Programa de Pós-graduação em Genética da Universidade Federal do Rio de Janeiro (PGGen-UFRJ), Rio de Janeiro, Brazil; 3 Laboratório de Genômica Aplicada e Bioinovações, Instituto Oswaldo Cruz, FIOCRUZ, Rio de Janeiro, Brazil; 4 Programa de Pós-graduação em Biomedicina Translacional, Universidade do Grande Rio/AFYA, Rio de Janeiro, Brazil; 5 Laboratório de Genética Humana, Instituto Oswaldo Cruz, FIOCRUZ, Rio de Janeiro, Brazil; 6 Programa de Pós-graduação em Biologia Celular e Molecular, Instituto Oswaldo Cruz, Fiocruz, Rio de Janeiro, Brazil; 7 ECLAMC no Departamento de Genética, Laboratório de Malformações Congênitas (LMC), Instituto de Biologia, Universidade Federal do Rio de Janeiro, Rio de Janeiro, Brazil

**Keywords:** adhesion protein, association study, genotyping, molecular modeling, NECTIN1 protein, orofacial cleft, sequence analysis

## Abstract

**Introduction:**

Orofacial cleft is a congenital anomaly influenced by genetic and environmental factors. *NECTIN1* encodes an adhesion protein critical for the adherens junctions and has been associated with orofacial clefts. This study aimed to investigate the contribution of *NECTIN1* to the etiology of NSCL/P in a population from Patagonia, Argentina, a region with a high prevalence of orofacial clefts.

**Methods:**

First, an association study was conducted to evaluate the relationship between orofacial clefts and two *NECTIN1* single nucleotide variants: rs3829260 (G > C) and rs7940667 (C > A). Genotyping of 132 affected families was performed. The transmission disequilibrium test was applied, and identified variants were analyzed *in silico* using VarSome and ClinVar. No statistically significant association was found for rs3829260 (G > C) or rs7940667 (C > A). Subsequently, all six exons of *NECTIN1* were sequenced in 116 probands. Molecular modeling was then performed to evaluate the functional impact on protein structure.

**Results:**

Three rare heterozygous variants were identified in five probands: two non-synonymous variants (p.(Arg199Gln) and p.(Gly44Ser)) and one synonymous variant p.(His394=). Molecular modeling suggested that p.(Arg199Gln) and p.(Gly44Ser) could locally impact the structural dynamics and glycosylation pattern.

**Conclusion:**

These findings show the potential involvement of *NECTIN1* in orofacial clefts and suggest that rare genetic variants may contribute to disease susceptibility.

## 1 Introduction

Orofacial clefts (OC) are among the most frequent congenital craniofacial anomalies and are typically classified as cleft lip with or without cleft palate (CL/P) or cleft palate only (CP). Non syndromic cleft lip with or without cleft palate (NSCL/P) arises from a multifactorial interplay of genetic and environmental factors (Dixon et al., 2011), and its long term care demands underline the considerable public health burden associated with these conditions (Wehby and Cassell, 2010).

Cellular adhesion molecules (CAMs), which are integral to the morphology of facial structures, are involved in several biological processes, including signaling, cell division, and migration (Cavallaro and Dejana, 2011). Certain CAMs, including E-cadherin and p120-catenin, have already been implicated in NSCL/P (Antiguas et al., 2022). Nectins, a family of Ca2+-independent CAMs, work by forming dimers and, subsequently, the connection between adjacent cells. Their association with the cytoskeleton is mediated by afadin and plays a crucial role in the formation of adherens junctions (Samanta and Almo, 2015).

Initially, the *NECTIN1* gene (chromossome 11:q23.3) was related to the cleft lip and palate-ectodermal dysplasia syndrome (CLPED1, OMIM: #225060) by Suzuki et al. (2000) who detected homozygous variants in patients with the syndrome. Sözen et al. (2001) was the first to associate *NECTIN1* with NSCL/P, observing a higher frequency of the p.(W185X) variant in individuals from the Cumaná city of Venezuela compared to controls. Additional variants have since been described in several populations (Table 1), and studies have begun to explore associations with common *NECTIN1* SNVs (Neiswanger et al., 2006). Yet, despite growing genetic evidence, little is known about how specific variants might affect Nectin-1 at the structural or functional level.

**TABLE 1:** Rare *NECTIN1* variants previous described in NSCL/P cases.

Reference	Variant	Population studied	Number of cases	Prediction*
Peng et al. (2016)	p.(Leu18Phe)	Taiwan	2 NSFL/P	likely benign
Sözen et al. (2009)	p.(V89M)	United States of America	2 NSFL/P	Ambiguous
Peng et al. (2016)	p.(Ser112Thr)	Taiwan	1 NSFL/P	likely benign
Oner and Tastan (2016)	p.(Ser174Thr)	Turkey	80 NSFL/P and 3 controls	likely pathogenic
Sözen et al. (2001)	p.(Trp185X)	Venezuela	14 NSFL/P and 1 control	–
Oner and Tastan (2016)	p.(Thr187Asn)	Turkey	80 NSFL/P and 3 controls	likely pathogenic
Sözen et al. (2009)	p.(T187=)	Australia	1 NSFL/P	–
Sözen et al. (2009)	p.(Arg199Gln)	Australia	4 NSFL/P and 3 controls	likely benign
Scapoli et al. (2006)	p.(Arg199Gln)	Italy	3 NSFL/P and 1 control	likely benign
Sözen et al. (2009)	p.(T206=)	Australia	1 NSFL/P and 1 control	–
Scapoli et al. (2006)	p.(Arg210His)	Italy	3 NSFL/P	likely benign
Scapoli et al. (2006)	p.(Arg212His)	Italy	1 NSFL/P	likely benign
Tongkobpetch et al. (2008)	p.(Val395Met)	Thailand	1 NSFL/P	Ambiguous
Scapoli et al. (2006)	p.(Asp424=)	Italy	1 NSFL/P	–

* Prediction made according to the AlphaMissense platform Cheng et al. (2023).

To help bridge this gap, we investigated the contribution of *NECTIN1* to NSCL/P in a familial cohort from Patagonia, Argentina, combining genetic analysis with three dimensional structural modeling to explore how identified variants might reshape key features of Nectin-1. This integrative approach adds a mechanistic dimension that remains largely absent in previous *NECTIN1* investigations. Our objectives were to test family based association for two previously studied SNVs, sequence all coding exons to identify rare variants, and model their potential structural consequences using established computational modeling approaches.

## 2 Methods

### 2.1 Sample selection

The study included a cohort of 132 NSCL/P-affected individuals and their unaffected mothers and/or fathers (72 trios and 60 dyads), a totaling of 336 biological samples was collected by the Latin American Collaborative Study of Congenital Malformations (ECLAMC) (Castilla and Orioli, 2004) in Patagonia, Argentina. This region, among others, was identified in a previous ECLAMC epidemiologic study as representing a high-prevalence geographic cluster for CL/P in Latin America and Amerindian ancestry was suggested as a risk factor for OCs (Poletta et al., 2007). All participants reside in cities belonging to the provinces of Rio Negro and Chubut. Families with at least one individual affected by OC were included in the study. Families that also had individuals affected by cleft palate were excluded due to the large number of syndromes segregating in families with this combination of orofacial clefts (Murray, 2002). In addition, individuals with dysmorphic syndromes that included CL/P as a clinical feature were excluded from this study to restrict the analysis to NSCL/P. The study protocol and informed consent form were approved by the Comité de Ética en Investigación del Centro de Educación Médica e Investigaciones Clínicas Dr Norberto Quirno in Buenos Aires, Argentina (IRB1745, IORG-0001315; approval number: #238). Prospective study patients and their families were informed about the nature of the study, and all subjects provided written informed consent before enrollment.

### 2.2 SNV selection, genotyping and statistical analysis for family-based association study

We selected two single-nucleotide variants (SNVs) with a minor allele frequency (MAF) ≥ 0.05 in the CEU population (Utah residents with Northern and Western European ancestry); rs3829260 (G > C) and rs7940667 (C > A) were previously reported in the literature as being associated with orofacial clefts (Neiswanger et al., 2006; Avila et al., 2006). rs3829260 (G > C) is located at intron 1 and rs7940667 (C > A) is missense and located at exon 6. Genotyping for rs3829260 (G > C) and rs7940667 (C > A) was performed by real-time PCR for a total of 336 individuals (132 cases and 204 unaffected mothers and/or fathers). We used chi-square calculations to assess Hardy-Weinberg equilibrium. The transmission disequilibrium test (TDT) was used to perform the familial association analysis. In brief, this test detects evidence of over-transmission of a particular allele from parents to affected offspring. All analysis was conducted using PLINK software (Purcell et al., 2007).

### 2.3 Sanger sequencing

We directly sequenced *NECTIN1* in 116 NSCL/P-affected individuals from the same sample previously described, using Sanger sequencing. In summary, primers for all the coding regions of *NECTIN1* were designed (Supplementary Material S1) and PCR reactions (Supplementary Material S2) were conducted in thermocycler ProFlex PCR System (Applied Biosystems, New Jersey, EUA). Sequenced was conducted at Instituto Oswaldo Cruz (IOC - RPT01A). Electropherograms were aligned with the reference sequence (ENST00000264025.8). We predicted the functional effects of these variants on the protein by using the Varsome (Kopanos et al., 2019) and AlphaMissense Cheng et al. (2023) and searching ClinVar (Landrum et al., 2018) for clinical significance. TraP-score was used to evaluate synonymous variants impact on the final mRNA transcript (Gelfman et al., 2017). In affected individuals carrying functional variants, parental samples were sequenced to determine whether the variants were *de novo* or segregated within the family.

### 2.4 Three-dimensional structural modeling of Nectin-1

The amino acid sequence of NECTIN1 was obtained from the UniProtKB database (accession Q15223). The Robetta protein structure prediction server (Kim et al., 2004) was used to construct a three-dimensional model of nectin-1 (residues 31–517) using the RoseTTAFold protocol, which employs deep learning algorithms for *de novo* protein structure prediction. Post-translational modifications including N-linked glycans, phosphorylated residues, and disulfide bonds were incorporated into the Nectin-1 model using the CHARMM-GUI server (Lee et al., 2016). To determine the homophilic binding mode of nectin-1, we performed structural alignment against the nectin-1 ectodomain crystal structure (PDB 4FMF) (Harrison et al., 2012) using the matchmaker command in UCSF ChimeraX (Pettersen et al., 2021). The positioning and tilt angle of nectin-1 relative to the lipid membrane bilayer were estimated using the Positioning of Proteins in Membrane (PPM) server (available online at https://opm.phar.umich.edu/ppm_server3_cgopm) Lomize et al. (2012). This computational approach utilizes an empirically adjusted, physically based implicit membrane model to predict the optimal insertion and orientation of membrane proteins. The p.(Gly44Ser) and p.(Arg199Gln) variants were introduced into the nectin-1 model using the swapaa command in ChimeraX, guided by Dunbrack backbone-dependent rotamer libraries (Dunbrack and Cohen, 1997). Electrostatic potential surfaces were visualized in ChimeraX. Solvent-accessible surface areas (SASA) were quantified using the GROMACS 2023.2 analysis tools (Abraham et al., 2015).

## 3 Results

### 3.1 Familial association analysis

A total of 105 families with cleft lip and palate and 27 families with cleft lip only were included in this study. Basic characteristics of the sample studied are shown in Table 2. The two SNVs rs3829260 (G > C) and rs7940667 (C > A) did not deviate from the Hardy-Weinberg equilibrium and none of the SNVs was associated with NSCL/P in this sample, the same result was found for the parent-of-origin effect (Supplementary Material S3).

**TABLE 2:** Sample characteristics.

Characteristic	n (%)
Number of families	132
Families with only one case	97 (73.5%)
Families with recurrence	35 (26.5%)
Female NSCL/P-affected	56 (42.4%)
Male NSCL/P-affected	76 (57.6%)
NSCLP-affected	105 (79.6%)
NSCL-affected	27 (20.4%)

NSCL/P, Non-syndromic cleft lip with or without cleft palate; NSCLP, Non-syndromic cleft lip and palate; NSCL, Non-syndromic cleft lip.

### 3.2 Screening for variants in NECTIN1

**FIGURE 1:**
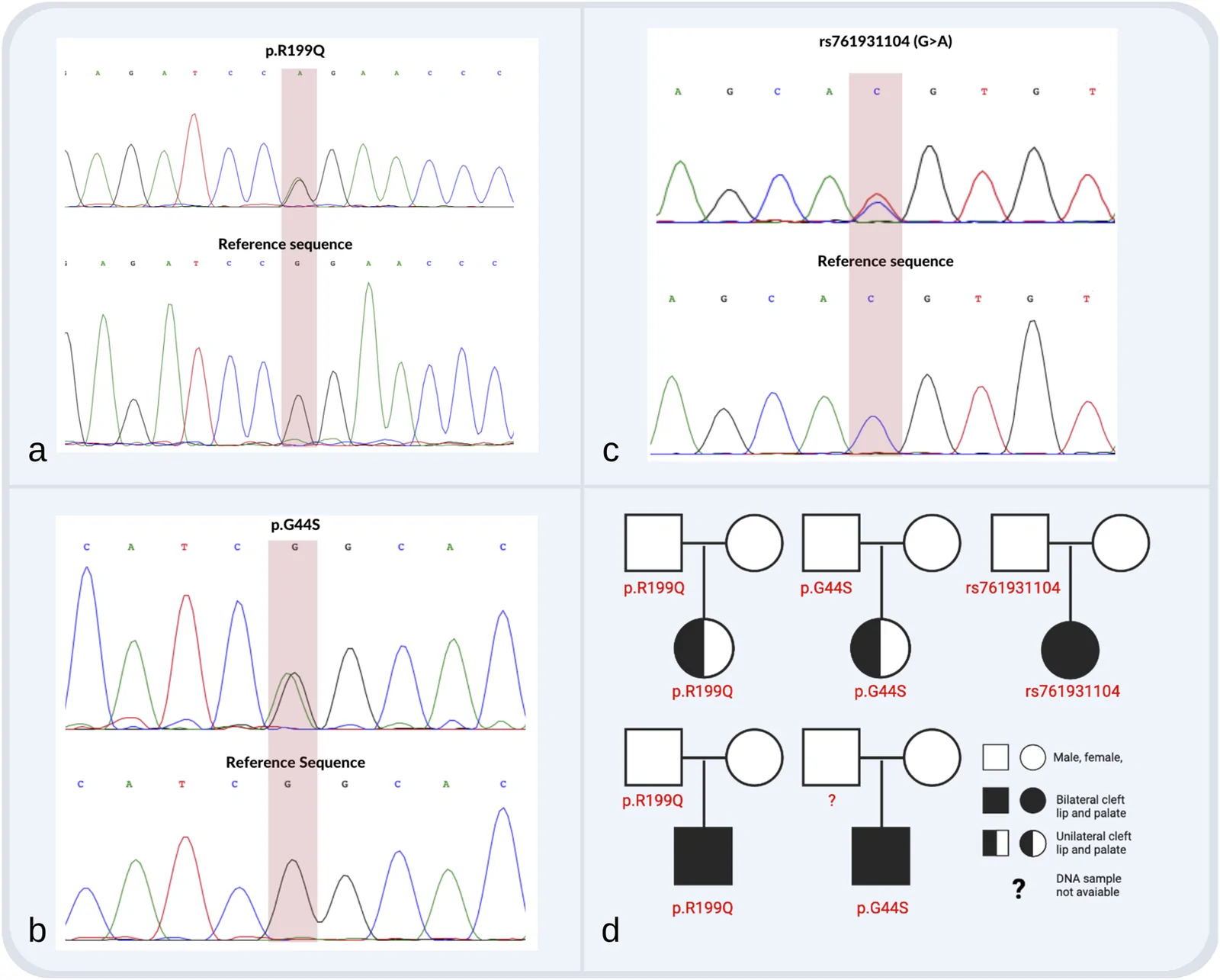
Molecular findings from the sequencing of *NECTIN1*. **(a)** Comparison between an electropherogram with p.(Arg199Gln) heterozygous variant and reference sequence. **(b)** Comparison between an electropherogram with p.(Gly44Ser) heterozygous variant and reference sequence. **(c)** Comparison between an electropherogram with rs761931104 (p.(His394=)) heterozygous variant and reference sequence. **(d)** Pedigree of the five families with variants in *NECTIN1*.

The first missense variant found was a change of arginine to glutamine at position 199 of the protein, caused by a substitution in exon 3 rs78809001, (NM_002855.5):c.596G > A | NP_002846.3:p.(Arg199Gln) (Figure 1a) and has a frequency in the gnomAD admixed american ancestry group of 0.0057. This variant was found in heterozygosity in two unrelated patients from the cities of Viedma and El Bolson (Rio Negro province), approximately 721 km apart, both families had recurrence of NSCL/P. The first patient was male, 13 years old, had bilateral NSCLP, and also had hair alteration, scoliosis, and absence of uvula and of the left canine tooth. The second patient was female, 2 years old, with unilateral NSCLP, dysarthria, left hypoacusis, and agenesis of the upper right lateral incisor tooth.

The second missense variant, detected in heterozygosity in exon 2 rs137991779, (NM_002855.5):c.130G > A | NP_002846.3:p.(Gly44Ser), was also found in two non-related patients from two cities 555 km apart (Viedma and Ingeniero Jacobaci, both in Rio Negro province), representing a glycine-to-serine substitution at position 44 (Figure 1b) and has a frequency in the gnomAD admixed american ancestry group of 0.00085. The first was a female, 1 year old with unilateral NSCLP and absence of an upper canine. The second, a male, 21 years old with bilateral NSCLP and a sharper and protruding chin according to the photos available.

Both variants were classified as benign by Varsome and had conflicting ClinVar interpretations (benign vs. uncertain significance), with some submissions referencing CLPED1 context. The Alphamisses platform classified p.(Arg199Gln) as likely bening and p.(Gly44Ser) as ambiguous. A final synonymous variant in exon 6 was found in one patient rs761931104, (NM_002855.5):c.1182C > T | NP_002846.3:p.(His394=) (Figure 1c), the TraP-score for this variant was 0.158 which is below the threshold for synonymous variants to be considered possibly damaging (0.221).

After the sequence of the genitors’ DNA it was possible to determine if the variants were inherited. The p.(Arg199Gln) variant was inherited in both cases from the father, also a heterozygous. The female patient with the variant in exon 2 (p.(Gly44Ser)) inherited from the father (heterozygous). In the case of the male patient, we only had DNA samples from the mother and we didn’t detect the variant. Finally, the variant in exon 6 was inherited from the mother, also heterozygous. Parents carrying the variants were evaluated for signs of microforms of OC and no such features were identified. A graphical resume of the findings is presented in Figure 1d.

### 3.3 Molecular modeling reveals local impacts of p.(Gly44Ser) and p.(Arg199Gln) variants on Nectin-1 structure

To elucidate the structural consequences of the p.(Gly44Ser) and p.(Arg199Gln) variants in nectin-1, we constructed an atomistic model covering the signal-cleaved ectodomain (residues 31–346), the single-pass transmembrane helix, and the short cytoplasmic tail, focusing analyses on the ectodomain. The ectodomain of nectin-1 comprises three Ig-like domains (V–C1–C2) that mediate cis/trans adhesion. N-linked glycosylation is expected at multiple asparagines within the ectodomain (e.g., Asn72, Asn139, and Asn202), as reported for nectin family members (Harrison et al., 2012). These regions are relevant to our study as the p.(Gly44Ser) and p.(Arg199Gln) variants are located within domains V and C1, respectively. The model showed the proximity of the variants to the key N-linked glycosylation sites Asn72, Asn139 (Supplementary Material S4).

**FIGURE 2:**
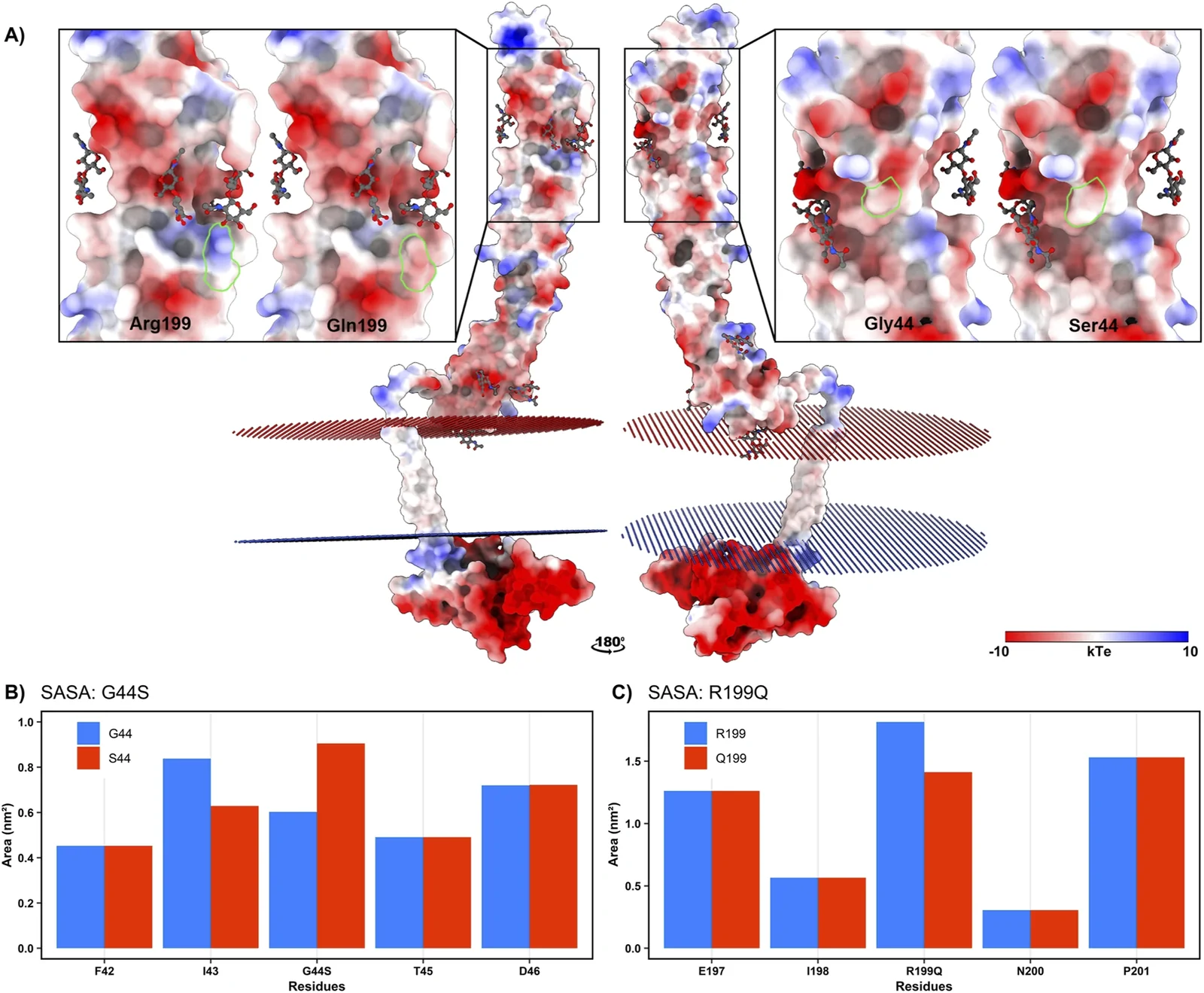
**(A)** Local electrostatic alterations in nectin-1 induced by p.(Gly44Ser) and p.(Arg199Gln) variants. Molecular surfaces of wild-type and mutant nectin-1 structural models are depicted with electrostatic potential coloring (red, acidic; white, neutral; blue, basic). Residues Gly44 and Arg199 subjected to variants are highlighted in green. The p.(Arg199Gln) substitution induces changes in the electrostatic landscape proximal to residue 199 in the C1 domain. In contrast, the p.(Gly44Ser) variant does not substantially alter the electrostatic surface near site 44. The predicted membrane topology orienting the extracellular and intracellular regions in red and blue, respectively. **(B,C)**Variant-induced alterations in residue solvent accessibility of nectin-1 structural models. Solvent accessible surface area (SASA) values are shown for **(B)** G44S - p.(Gly44Ser) and **(C)** R199Q - p.(Arg199Gln) variants, with wild-type and mutant models depicted in blue and red, respectively. Introduction of the larger serine side chain at position 44 leads to increased SASA in this residue. The p.(Gly44Ser) variant also decreases SASA of the neighboring isoleucine 43 residue. In contrast, the p.(Arg199Gln) variant decreases SASA at position 199, leaving neighboring residues unchanged.

Regarding the p.(Arg199Gln) variant, the divergence in biochemical properties between arginine and glutamine was investigated. Arginine has a guanidino group in its side chain, which, under physiological conditions (pH 7.4), has a positive charge due to its nitrogen content. This charge enables arginine to promote hydrogen bonding and electrostatic (charge-charge) interactions, particularly with negatively charged groups. Conversely, glutamine, an uncharged polar amino acid, has an amide group on its side chain which allows hydrogen bonding interactions but does not have the electrostatic interaction capability of arginine. Computational analysis predicted that the p.(Arg199Gln) variant would significantly alter the electrostatic potential landscape of nectin-1. In the wild-type model, the region around Arg199 had a predominantly positive potential. However, the p.(Arg199Gln) mutant showed a negative electrostatic potential at the same position (Figure 2A). Furthermore, Gln199 was located 6.2 Å and 7.5 Å away from the Asn72 and Asn139 glycans, respectively. Focusing on the p.(Gly44Ser) variant, the contrasting physicochemical properties of glycine and serine were investigated. Glycine, the smallest amino acid, has exceptional flexibility due to its unique hydrogen atom side chain. Conversely, serine, a polar amino acid, has a hydroxyl group (-OH) in its side chain which allows it to form hydrogen bonds with other molecules. Notably, the p.(Gly44Ser) variant did not induce any changes in the electrostatic potential pattern (Figure 2A), and Ser44 was found to be approximately 6.5 Å away from the glycan of Asn-202.

Quantitative calculations of per-residue SASA computed with gmx sasa (probe 0.14 nm) showed that the p.(Gly44Ser) variant increased the SASA from 0.603 nm^2^ (Gly) to 0.905 nm^2^ (Ser). Notably, this variant also decreased the SASA of the adjacent Ile43 residue from 0.838 nm^2^ to 0.629 nm^2^, indicating possible local structural perturbations (Figure 2B). In contrast, the p.(Arg199Gln) variant showed a reduction in area from 1.814 nm^2^ to 1.412 nm^2^, leaving the neighboring residues unchanged (Figure 2C).

## 4 Discussion

In this study, we analyzed a familial cohort from a region with a high prevalence of orofacial clefts. No association was found between two common *NECTIN1* single nucleotide variants (SNVs) and NSCL/P. The rs3829260 (G > C) variant was previously associated with NSCL/P in a sample of 25 extended families from Guatemala (Neiswanger et al., 2006). However, subsequent studies by Avila et al. (2006) and Cheng et al. (2012) found no association in cohorts from Denmark, Iowa, the Philippines, Latin America and China, respectively. Similarly, rs7940667 (C > A) yielded conflicting results - being associated with cleft lip/palate (CL/P and CP) in samples from the Philippines, Denmark, and Iowa, but not replicated in a Brazilian case-control study by Paranaíba et al. (2013). More recently, Park et al. (2024) reported evidence of parental over transmission in Asian and European populations. These discrepancies may reflect population-specific genetic backgrounds and highlight the importance of studying diverse cohorts.

Focusing on rare variant screening, we identified three variants in *NECTIN1* exons. The first, p.(Arg199Gln) in exon 3, was found in heterozygous form in two NSCL/P cases and was inherited paternally. In the 1000 Genomes Project, the Colombian population showed the highest frequency of this variant in Latin America (1.6%). This variant has already been found in three NSCL/P-affected individuals and 2 controls by Scapoli et al. (2006). One patient also had hair alterations, hypodontia and absence of uvula, features that may be related to CLEPD1. The other variant was detected in exon 2 (p.(Gly44Ser)), also found in heterozygosis in two cases and not previously reported in NSCL/P-affected individuals. The 1000 Genomes Project showed a frequency of 1,1% in the Colombian population. One of our patients with p.(Gly44Ser) variant also presented a prominent and flattened chin. Surprisingly, the study of Liu et al. (2021) correlated the presence of variants in *NECTIN1* with a sharper and protruding chin. Research has previously suggested that facial features may act as predisposing factors for orofacial cleft, based on the fact that there are distinct orofacial features in parents of patients with orofacial cleft compared to parents of controls, such as, retrusion of the jaw (Weinberg, 2022).

We highlight the limitations of a heterozygous variant, but point out that NSCL/P is a complex anomaly with both genetic and environmental components. Also, the presence of variants unaffected individuals could be explained by other genetic and environmental factors that could imply in a higher risk for developing NSCL/P and influence the penetrance of this variant as shown by Yu et al. (2022). Other limitation of the present study is the absence of a locally recruited control cohort for the sequencing analysis. Variant frequencies were instead compared with public reference panels (1000 Genomes Project, gnomAD), using the Latino/Admixed American superpopulation as the closest available proxy for our Patagonian sample. These databases, however, do not fully represent this region. Future studies with a matched, locally recruited control cohort are needed to address this limitation.

Nectin-1 mediates cell-cell adhesion through homophilic and heterophilic trans-interactions dependent on its N-terminal V-domain (Samanta and Almo, 2015). Crystallographic analysis and mutagenesis studies have revealed a canonical dimerization mode involving the C’C″CFG β -sheet Di Giovine et al. (2011); Narita et al. (2011); Yasumi et al. (2003). Critical residues at this interface include Phe129, which intercalates into a hydrophobic pocket on the partner molecule, and supporting contacts from Thr63, Asn77, and Met85 (Di Giovine et al., 2011; Martinez and Spear, 2002; Struyf et al., 2002). Variant of these residues impairs nectin-1 binding and function, highlighting their importance.

Our structural analysis focused on the potential impact of the p.(Gly44Ser) variant on this dimerization interface. Gly44 is located on the FG loop in close proximity to, but not directly at, the binding site (Di Giovine et al., 2011). The serine substitution could affect the interactions through local steric or dynamic effects. Although not a binding site residue, Gly44 provides flexibility that may allow movement of nearby side chains critical for interactions. Rigidity caused by the p.(Gly44Ser) variant could restrict these movements and reduce affinity, as has been shown for other adhesion molecules (Vendome et al., 2014). In addition, the introduced serine hydroxyl may form novel hydrogen bonds that propagate subtle changes in the binding surface. Given its location within the V-domain, Gly44 lies adjacent to the Asn72 glycosylation region rather than Asn202; proximity to Asn72 could, in principle, modulate local water/glycan packing without implying altered glycosylation efficiency.

Additionally, we analyzed the potential impact of the p.(Arg199Gln) variant located in the C1 domain of nectin-1. Although not directly participating in binding, the C1 domain contributes to optimal positioning of the V-domain, cis/trans geometry, receptor clustering, and downstream signaling Samanta and Almo (2015); Takai et al. (2008). Thus, variants in C1 could indirectly impair interactions and function. Arg199 is located near the V-C1 elbow region important for proper domain orientations. The p.(Arg199Gln) substitution would markedly alter the electrostatic potential, eliminating the positive charge of arginine’s guanidino group. Electrostatic potential calculations predicted that this variant induces a negative potential in this region, which could disturb local protein-protein or protein-glycan interactions. Furthermore, Arg199 is close to N-linked glycans at Asn72, Asn139 and Asn202. Although Arg199 is not part of a canonical N-X-S/T sequon, the loss of its positive charge in the immediate environment of these glycans could modulate local protein–glycan contacts. Aberrant glycosylation can impact protein stability and function (Scott et al., 2011), but functional validation, such as cell-based adhesion assays to directly test the impact of p.(Arg199Gln) and p.(Gly44Ser) on Nectin-1 binding capacity, represents an important next step to confirm the structural predictions reported here.

The SASA analysis complements these observations by quantifying how each substitution reshapes the local solvent-exposed surface. Solvent accessibility determines which side chains are available for hydration, hydrogen bonding, electrostatic screening and intermolecular contacts, and therefore delimits where a variant can plausibly exert an effect. For p.(Gly44Ser), the increased exposure at position 44 indicates that the introduced hydroxyl group is oriented towards the solvent, while the concomitant burial of Ile43 shows that this gain is redistributive rather than additive, reflecting a local change in surface polarity and packing at the periphery of the dimerisation interface. For p.(Arg199Gln), the reduced exposure at position 199 accompanies the loss of the guanidino positive charge, indicating a decrease in both exposed surface and charge density at the V–C1 elbow, which may influence domain orientation and the electrostatic environment sampled by the nearby glycans and by potential interaction partners. Importantly, in both variants the SASA of the flanking residues remained unchanged, indicating that the perturbation is confined to the substituted position and, for p.(Gly44Ser), to its immediate neighbour. Since SASA is a geometric descriptor derived from static models, it does not quantify thermodynamic stability or conformational dynamics; the present data therefore support localized modulation of nectin-1 surface properties near functionally relevant regions, rather than global destabilization, and a formal assessment of stability and dynamics would require free-energy calculations and molecular dynamics simulations.

In summary, the p.(Arg199Gln) and p.(Gly44Ser) variants may exert localized effects on the Nectin-1 structure, dynamics, and glycosylation pattern near functionally important regions involved in adhesive binding and signaling. Overall, our integrative approach suggests that *NECTIN1* rare variants—rather than common variants—may contribute to NSCL/P susceptibility in this Patagonian cohort. These results also illustrate the value of combining sequencing, familial segregation analyses, phenotype review, and structural modeling to clarify the potential functional relevance of low frequency coding variation.

This study found no evidence of association between two common *NECTIN1* SNVs and NSCL/P in a high prevalence Patagonian cohort. In contrast, sequencing identified two rare heterozygous missense variants, p.(Gly44Ser) and p.(Arg199Gln), with molecular modeling supporting localized structural effects compatible with subtle modulation of adhesion or domain orientation rather than global protein destabilization. Although not sufficient to establish causality, these findings underscore the potential contribution of rare *NECTIN1* variants to the multifactorial risk architecture of NSCL/P. Taken together with their rarity in population databases, recurrence in unrelated affected individuals, and prior reports in independent cohorts, these variants are best interpreted as candidate low-penetrance risk alleles rather than individually causal mutations. Future studies incorporating larger cohorts, quantitative facial phenotyping, and functional assays will be essential to elucidate the specific biological mechanisms through which *NECTIN1* variation influences orofacial cleft susceptibility.

## Data Availability

The original contributions presented in the study are publicly available. This data can be found in the ARCA Dados repository with the accession number https://doi.org/10.35078/SPF4P3.
