# Supplementary material for: Rare missense variants in NECTIN1 alter local protein structure and may contribute to non-syndromic cleft lip with or without palate
Source: Front Genet. 2026 Aug 13;17:1894075. doi: 10.3389/fgene.2026.1894075 (PMC13518071; doi:10.3389/fgene.2026.1894075)
Supplement: Supplementary file 1 [file DataSheet1.pdf]

Supplementary Material

1 TABLE S1: PRIMER SEQUENCES DESIGN TO AMPLIFY SIX EXONS OF *NECTIN1*

Exon	Primer sequence (5' → 3')	Fragment size (bp)	Tm (°C)
1F	CGCTCTGCGTGGAAGAAG	559	55
1R	GTTCGGCTTCTCAACCTCTTT		
2F	TGGGATGTTGTCACTTTCTTC	698	59
2R	TGGCTAGAAGGGACTGGAA		
3F	GGTTCTGGGACAGTCTTTGG	697	61
3R	ATGGAAACAGCCAGGAGAGA		
4F	TCCTCCCTTTCTCTTTCTGG	596	61
4R	GCTTGTTCCATTGACCTTGA		
5F	CCTCAGGTTTCATCTGTTAAGG	667	55
5R	GCACTTCCTTCTTCCCTCTC		
6AF	TCCTCTTGCCTGTCTCTCAC	698	65
6AR	AGCTGCTCAGGGTCGTACT		
6BF	ATCCTGCTGGTGTTGATTGT	647	57
6BR	AAAACACAAAGCCAAGTCGT		

bp: base pairs; Tm: annealing temperature

2 PCR CONDITIONS

PCR conditions: 15.7μL ultrapure water, 2.5μL of reaction buffer (10x), 2.5μL of dNTPs (2mM), 0.5μL of forward and reverse primer, 1μL de MgCl2 (50mM), 0.3μL of Platinum Taq DNA polymerase (Invitrogen, Massachusetts, EUA) was prepared. We added 23μl of the master mix to 2μL of DNA (30ng/μL). PCR conditions were as follows: 94°C for 2 minutes, 35 cycles of 94°C for 30 seconds, annealing temperature for 30 seconds and 72°C for 1 minute.

3 RESULTS FROM THE ASSOCIATION TEST BETWEEN COMMON SNVS IN NECTIN1 AND NSCL/P

SNV	HW	MAF	p-value	OR (95% CI)	p-mat	p-pat
rs7940667	0.081	0.041	0.705	0.75 (0.17–3.35)	0.317	0.563
rs3829260	0.081	0.344	0.884	0.96 (0.54–1.7)	0.857	1

SNV: Single nucleotide variants; HW: result from Hardy-Weinberg equilibrium test; MAF: minimum allele frequency; OR: odds ratio; p-mat: p-value for maternal transmission; p-pat: p-value for paternal transmission.

4 FIGURE

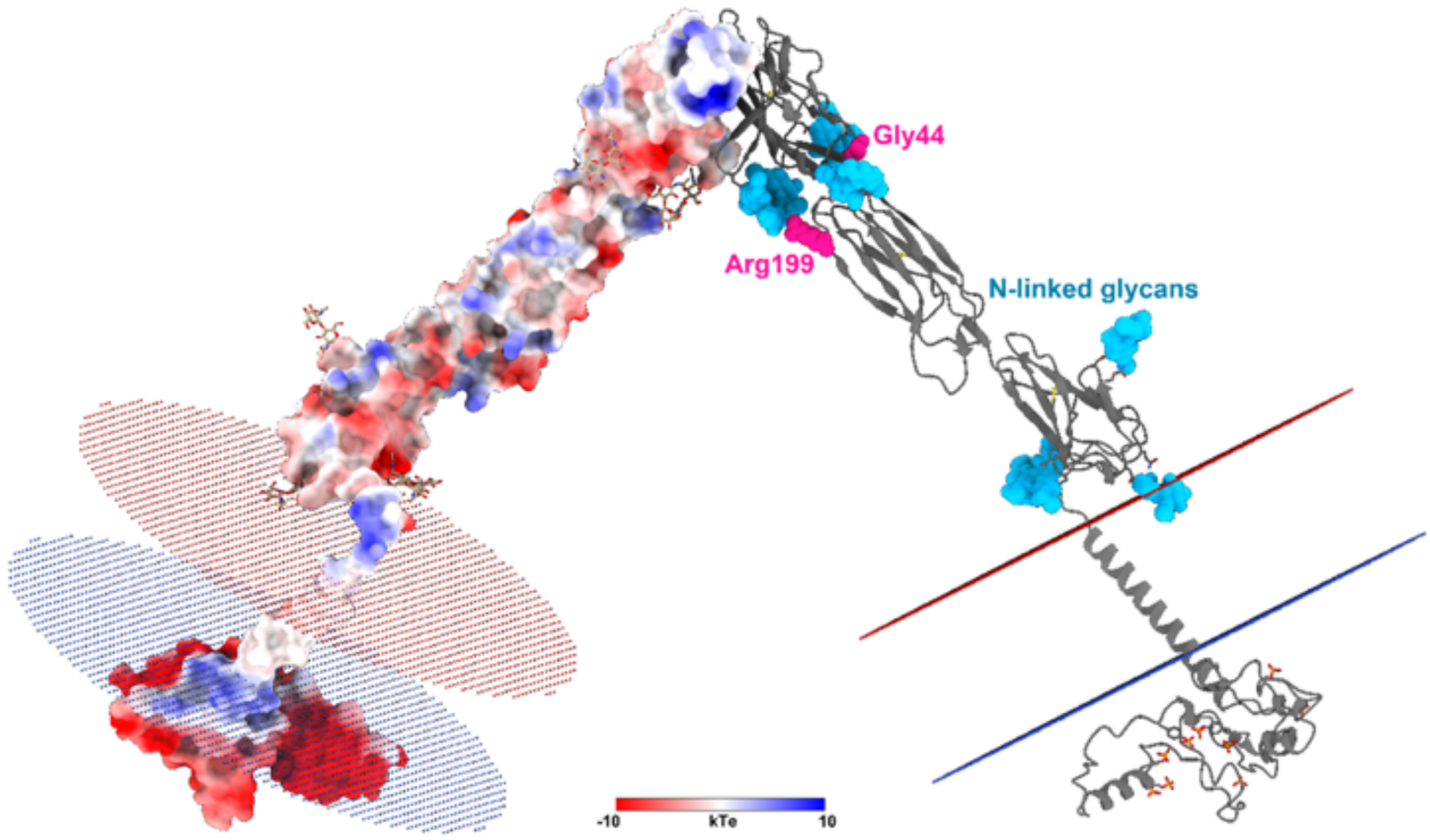

Figure S1. Structural model of the nectin-1 homodimer interface. The molecular surface of one nectin-1 monomer is depicted as colored by electrostatic potential (red, acidic; white, neutral; blue, basic). The second monomer is shown in ribbon representation with variants regions Gly44 and Arg199 highlighted as pink spheres. The ectodomain model includes 8 N-linked glycans (sticks). Phosphorylated residues in the intracellular region are displayed in stick representation. The predicted membrane orientation is shown with extracellular and intracellular regions in red and blue, respectively.
